# Functional diversity of jasmonates in rice

**DOI:** 10.1186/s12284-015-0042-9

**Published:** 2015-01-29

**Authors:** Zheng Liu, Shumin Zhang, Ning Sun, Hongyun Liu, Yanhong Zhao, Yuling Liang, Liping Zhang, Yuanhuai Han

**Affiliations:** College of Life Sciences, Hebei University, Baoding, China; The Affiliated School of Hebei Baoding Normal, Baoding, China; College of Agriculture, Ludong University, Yantai, China; School of Agriculture, Shanxi Agricultural University, Taigu, Jinzhong, China; Key Laboratory of Crop Gene Resources and Germplasm Enhancement on Loess Plateau, Ministry of Agriculture, Taiyuan, China

**Keywords:** Jasmonate, Growth and development, Environmental and abiotic responses, Pest and pathogen resistance, Hormone crosstalk

## Abstract

Phytohormone jasmonates (JA) play essential roles in plants, such as regulating development and growth, responding to environmental changes, and resisting abiotic and biotic stresses. During signaling, JA interacts, either synergistically or antagonistically, with other hormones, such as salicylic acid (SA), gibberellin (GA), ethylene (ET), auxin, brassinosteroid (BR), and abscisic acid (ABA), to regulate gene expression in regulatory networks, conferring physiological and metabolic adjustments in plants. As an important staple crop, rice is a major nutritional source for human beings and feeds one third of the world’s population. Recent years have seen significant progress in the understanding of the JA pathway in rice. In this review, we summarize the diverse functions of JA, and discuss the JA interplay with other hormones, as well as light, in this economically important crop. We believe that a better understanding of the JA pathway will lead to practical biotechnological applications in rice breeding and cultivation.

**Electronic supplementary material:**

The online version of this article (doi:10.1186/s12284-015-0042-9) contains supplementary material, which is available to authorized users.

## Introduction

Jasmonates (JA) are a class of polyunsaturated fatty acid-derived phytohormones, playing important roles in plant growth and defense responses. The biosynthesis of JA initiates in chloroplasts, involving the release of α-linolenic acid (α-LeA, 18:3 or 18:2) from the lipid membrane by phospholipases (PLDs). Only α-LeA (18:3) is utilized as a JA precursor through one of the seven distinct branches of the lipoxygenase (LOX) pathway, the allene oxide synthase (AOS) branch. Traditionally, α-LeA (18:3) is oxidized by 13-LOX, an enzyme catalyzing regio- and stereo-specific dioxygenation of polyunsaturated fatty acid at C-13, to specifically form a fatty acid hydroperoxide, 13-HPOT (13*S-*hydroperoxy-(9*Z*,11*E*,15)-octadecatrienoic acid); however, the generation of both 9- and 13-hydroperoxides by OsLOX1 based on a dual C-9 and C-13 specificity was found in rice (Wang et al. 2008). The 13-HPOT then enters the seven enzymatic branches of the LOX pathway, including the oxidation by AOS to form an allene oxide, 12,13-EOT ((9*Z*,13*S*,15*Z*)-12,13-oxido-9,11,15-octadecatrienoic acid). The 12,13-EOT is unstable and can be cyclized by allene oxide cyclase (AOC) to form racemic 12-oxophytodienoic acid (12-OPDA). Subsequently, the cyclized OPDA is transferred from chloroplasts into peroxisomes, where it is reduced by OPDA reductase3 (OPR3) and three further β-oxidation steps are conducted to produce (3*R*, 7*S*)-(+)-JA. After being released into the cytosol, it is converted into (3*R*, 7*R*)-(−)-JA, which can then be catalyzed by a jasmonate-amido synthase, JASMONATE RESISTANT 1 (JAR1), to form bioactive jasmonate (JA-Ile) by conjugating the amino acid isoleucine to (3*R*, 7*R*)-(−)-JA (Lyons et al. 2013; Svyatyna and Riemann 2012; Schaller and Stintzi 2009). Additionally, the hydroperoxy-octadecadienoic acids (HPOTs/HPODs) generated by LOXs can enter the hydroperoxide lyase (HPL) branch of the LOX pathway and are finally converted to green leaf volatiles (GLVs), which are indirectly involved in the defense against herbivores. Thus, the AOS and HPL branches compete for the same substrates and play antagonistic actions in rice (Chehab et al. 2007; Lyons et al. 2013).

**Figure 1:**
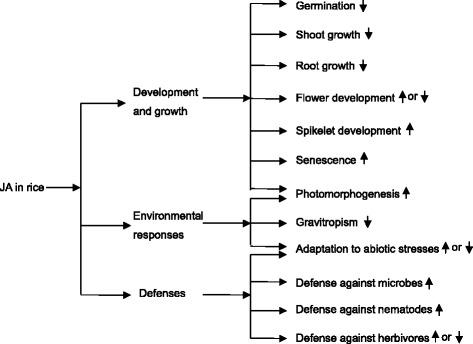
**Diverse functions of jasmonates (JA) in rice.** Upwards arrows represent positive regulation and downwards arrows represent negative regulation by JA.

JA activates a succession of signaling pathways, resulting in the activation of the genes required for diverse functions, such as the regulation of plant growth and development (Browse 2009a,b), mediation of biotic and abiotic resistances (Browse 2009a, Santino et al. 2013), responses to different environmental conditions (Svyatyna and Riemann 2012), and crosstalk with other phytophormones (Vleesschauwer et al. 2013). Although most research on JA in plants was conducted in the model dicot plant *Arabidopsis thaliana*, significant progress has been made in recent years in rice (*Oryza sativa*), a monocot that is an important staple crop worldwide. Here, we review the diverse functions of JA in rice growth and development, environmental and abiotic responses, and pest and pathogen resistance as shown in Figure 1. Additionally, we highlight the importance of the interplay between JA and other hormones, as well as light, in rice. A functional comparison of the genes responsible for JA biosynthesis and signaling between rice and other plants, especially *Arabidopsis* is also provided (Table 1).

**Table 1:** **Comparison of genes responsible for Jasmonates (JA) biosynthesis and signaling between rice and other plants**

**Gene**	**Functions**	**References**	**Species**	**Gene**	**Functions**	**References**
*TASSELSEED-2*	Increases contents of JA precursors, resulting in sterility	Liu et al. 2012a	maize	*TASSELSEED-1*	Lipoxygenase, affecting JA signaling in sex determination	Acosta et al. 2009
*OsAOS*	JA biosynthesis; ^1^sterility when silenced; ^2^resistance to *Xanthomonas oryzae* pv. *oryzae* (*Xoo*) and *Magnaporthe grisea* when overexpressed; ^3^activated by red and blue light	^1^Bae et al. 2010; ^2^Mei et al. 2006; ^3^Haga and Iino 2004	^*1,2*^ *Arabidopsis*	*AOS*	^1^JA biosynthesis; ^1^sterility when mutated; ^2^susceptible to bacterium *Erwinia carotovora* ssp. *carotovora* strain WPP14 (*Ecc*) when mutated; ^3^susceptible to *Mycosphaerella pinodes* when silenced	^1^Park et al. 2002; ^2^Pajerowska-Mukhtar et al. 2008; ^3^Toyoda et al. 2013
			^3^ *Pea*			
*OsOPR*	^1^JA biosynthesis; sterility complement when expressed in *Arabidopsis opr3* mutant; ^2^activated in response to red light	^1^Tani et al. 2008; ^2^Riemann et al. 2003	^*1*^ *Arabidopsis*	*OPR3*	^1^JA biosynthesis; ^1^sterility when mutated; ^2^susceptible to *M. pinodes* when silenced	^1^Tani et al. 2008; ^2^Toyoda et al. 2013
			^2^ *Pea*			
*OsAOC*	^1^JA biosynthesis; ^1^early flowering, elongated sterile lemma and reduced fertility; ^1^enhanced fungal hyphal growth when mutated; ^2^involved in light-mediated inhibition of coleoptile elongation	^1^Riemann et al. 2013; ^2^Riemann et al. 2003	^1^ *P. Patens*	^1^ *PpAOC*	^1^Reduced fertility and altered sporophyte morphology when mutated; ^2^susceptible to *M. pinodes* when silenced	^1^Stumpe et al. 2010; ^2^Toyoda et al. 2013
			^2^ *Pea*			
*OsJAR1*	^1^Catalyzing JA-Ile production to resist blast attack; ^1^coleoptiles grow longer when mutated under FR; ^2^functioning in JA-light signaling	^1^Wakuta et al. 2011; ^2^Riemann et al. 2008	*Arabidopsis*	*JAR1*	^1^Affecting root elongation and seed germination; ^2^increased susceptibility to the soil fungus *Pythium irregulare* when mutated; ^3^hypocotyl growth inhibition when mutated; ^4^required for shade responses	^1^Staswick et al. 1992; ^2^Staswick et al. 1998; ^3^Chen et al. 2007; ^4^Robson et al. 2010
*OsJMT1*	Promoting production of MeJA	Kim et al. 2009	*Arabidopsis*	*AtRMC*	Promoting production of MeJA; grain yield reduction when overexpressed in rice	Kim et al. 2009
*OsHI-LOX*	^1^JA biosynthesis; ^2^susceptible to rice striped stem borer (SSB) and resistant to rice brown planthopper (BPH) when knocked down	^1^Li et al. 2013; ^2^Zhou et al. 2009	*Arabidopsis*	*13-LOXs*	^1^Contributing to rapid jasmonate synthesis in wounded leaves; ^2^defense against herbivores	^1^Chauvin et al. 2013; ^2^Chauvin 2014
*OsCOI1*	^1^A JA receptor; ^1^faster germination when silenced; ^2^activating JA responsive genes; ^3^resistance to *Xoo*	^1^Yang et al. 2012; ^2^Katsir et al. 2008; ^2^Yamada et al. 2012; ^3^Yang 2009	*Arabidopsis*	*COI1*	^1^Male sterility when mutated; resistance to insect herbivory and pathogens; growth inhibition; ^2^required for light-induced suppression of hypocotyl elongation in response to both R and FR light; ^2^suppressing chlorophyll biosynthesis	^1^Browse 2009b; ^2^Robson et al. 2010
*OsJAZ*	^1^Functioning in JA signaling; ^1^regulating JA responsive gene; ^2^participating in drought resistance; ^3^ involved in JA-induced resistance to bacterial blight	^1^Toda et al. 2013; ^2^Seo et al. 2011; ^3^Yamada et al. 2012	*Arabidopsis*	*JAZ1*	^1^functional in shade responses; ^2^ *35S:JAZ1D* transgenic plants display male sterility, reduced defense against *Pseudomonas syringae*, and showing altered JA-mediated root growth inhibition; ^3^ displaying reduced resistance to the generalist herbivore *Spodoptera exigua*	^1^Robson et al. 2010; ^2^Thines et al. 2007; ^2^Melotto et al. 2008; ^3^Chung et al. 2008
*rim1*	^1^Negatively regulating JA signaling; ^1^root inhibition when mutated; ^2^confering resistance to *Rice dwarf virus*	^1^Yoshii et al. 2010; ^2^Yoshii et al. 2008	*Arabidopsis*	*ANAC028*	Unknown function	Yoshii et al. 2008
*OsWRKY45-1; OsWRKY45-2*	Positively regulating JA signaling; directing defense against *Xoo*	Tao et al. 2009	*Arabidopsis*	*AtWRKY45*	^1^JA responsive; ^2^positively regulating Pi uptake	^1^Schluttenhofer et al. 2014; ^2^Wang et al. 2014
*OsWRKY13*	suppressing JA signaling; defense against *Xoo* and *M. grisea*	Tao et al. 2009	*Soybean*	*GmWRKY13*	increased sensitivity to salt and mannitol stresses, increase in lateral roots when overexpressed in *Arabidopsis*	Zhou et al. 2008
*OsWRKY30*	^1^Activating JA biosynthetic pathway; ^1^defense against *Rhizoctonia Solani* and *M. Grisea*; ^2^confering drought tolerance	^1^Peng et al. 2012; ^2^Shen et al. 2012	*Capsicum annuum*	*CaWRKY30*	Induced by various pathogens and SA, suppressed by JA	Zheng et al. 2011
*OsNPR1*	^1^Suppressing JA signaling; ^1^resisting against SSB when knockdown; ^1^mediating JA and ET crosstalk; ^2^mediating JA and SA crosstalk	^1^Li et al. 2013; ^2^Yuan et al. 2007	*Arabidopsis*	*NPR1*	^1^Involved in the SA-mediated suppression of JA signaling; ^2^required for systemic resistance conferred by the Mycorrhizal fungus *Piriformospora indica*	^1^Spoel et al. 2003; ^2^Stein et al. 2008
*OsMPK*	Causing reduced levels of JA and susceptible to SSB larvae when silenced	Wang et al. 2013	*Arabidopsis*	*MPK4*	^1^Positive regulator of ET- and JA-mediated defenses; ^2^negative regulator of SA-dependent systemic required resistance; ^3^implicated in responses to abiotic stress	^1^Brodersen et al. 2006; ^2^Petersen et al. 2000; ^3^Ichimura et al. 2000
*OsPLDs*	Resistance to SSB infestation; reducing their expression resulted in the reduced elicited levels of linolenic acid, JA, green leaf volatiles, and ethylene	Qi et al. 2011	*Arabidopsis*	*PLDs*	^1^mediating wound induction of JA; ^2^required for high salinity and water deficit tolerance; ^3^functional in microtubule organization, ^4^salicylic acid signaling and ^5^seedling development	^1^Wang et al. 2000; ^2^Bargmann et al. 2009; ^3^Gardiner et al. 2003; ^4^Krinke et al. 2009; ^5^Motes et al. 2005

## Review

### Rice growth and development regulated by JA

#### Sterility

Flower development and sterility are effected by JA in plants, including *Arabidopsis*, tomato and maize (Wasternack and Hause 2013). In rice, a mitochondrial proteomic comparison between a sterile line and its fertile near-isogenic line revealed that a sex determination protein TASSELSEED-2 (Os07g46920.1) was significantly up-regulated in the sterile line. At the same time, JA precursors were significantly increased in all of the developmental stages of the reproductive organs in the sterile line, except at the bicellular pollen stage, indicating that the biosynthetic levels of JA may regulate rice sterility (Liu et al. 2012a). The maize homologue of TASSELSEED-1 affects JA signaling, further confirming the effects of JA on rice flower development (Acosta et al. 2009). More direct evidence of JA functioning in rice sterility has been revealed by studying rice genes that are responsible for JA biosynthesis. Similar to the *AOS* knock-out mutants of *Arabidopsis* (Park et al. 2002), *OsAOS1* and *OsAOS2* RNAi-silenced transgenic rice plants show a severe or complete sterility phenotype during the reproductive stage (Bae et al. 2010). The rice *OsOPR7* gene that is functionally similar to *Arabidopsis opr3*, which is involved in JA biosynthesis, is able to complement the male sterility phenotype of *Arabidopsis opr3* mutants and restores JA production (Tani et al. 2008). Further, JA-deficient mutants, with a disrupted expression of *OsAOC* also show typical developmental phenotypes such as early flowering, elongated sterile lemma, and reduced fertility (Riemann et al. 2013). The over-production of JA is also thought to affect rice fertility. The oxylipin pathway contains several competing branch pathways, including allene oxide synthase (AOS) and hydroperoxide lyase (HPL), which are responsible for the production of JAs and aldehydes, respectively. Disruption of the rice HPL pathway by mutations results in a dramatic increase in JA production, leading to a reduced seed-setting ratio and a reduced tiller number due to the interference of pollen fertility (Liu et al. 2012b). These results indicate that JA plays critical roles in rice flower development and fertility.

#### Seed germination

Many factors participate in seed germination, including plant hormones, which play critical roles. Gibberellins (GAs), abscisic acid (ABA), auxin, ethylene (ET), cytokinins, and brassinosteroids (BRs) function during seed germination. For example, because of the effects of antagonism between GA and ABA, seeds are released from dormancy (Ye and Zhang 2012). Although no direct effect of auxin on seed germination has been reported, some AUXIN RESPONSE FACTORS are involved in seed germination. It was found that the inhibition of AUXIN RESPONSE FACTOR 10 by microRNA60 is necessary for seed germination (Liu et al. 2007). An increasing amount of ET was also reported during seed germination in different plant species and that ET can release seed dormancy (Borghetti et al. 2002). Cytokinins promote seed germination by alleviating stresses (Miransari and Smith 2014), and seed germination can be enhanced by BR because it offsets the negative effects of ABA on germination (Zhang et al. 2009). JA has long been proposed to inhibit seed germination. Wilen et al. (1991) reported that exogenous applications of JA affected embryo-specific processes in *Brassica* and *Linum* oilseeds. In recent years, it was further revealed that OPDA, a key metabolic intermediate during JA biosynthesis, has a synergistic effect with ABA in germination inhibition (Dave et al. 2011). In rice, some evidence indicates that JA also regulates seed germination negatively. The imbibition of a MeJA solution by rice seeds produced a significant inhibition of germination (Tang et al. 2002). The overexpression of pepper MAP kinase, which resulted in a high accumulation of JA, in transgenic rice inhibited seed germination (Lee and Back 2005) and the silencing of the rice *Coronatine Insensitive 1* (*OsCOI1*) gene, a key component of a JA receptor, led to a faster germination compared with wild-type rice plants (Yang et al. 2012). It should be noted that studies on functions of JA during rice seed germination are still preliminary and efforts should be taken to unravel the mechanisms of germination that are regulated by JA in rice.

#### Root growth inhibition

Auxin plays an essential role during lateral root (LR) development by establishing an auxin gradient mediated by PIN-FORMED2 (PIN2), an auxin efflux transporter (De Smet et al. 2006). However, recent studies revealed that by interacting with auxin and affecting auxin biosynthesis, JA fine-tunes the regulation of LR formation. In fact, an efficient method to identify JA-related mutants in *Arabidopsis* is by observing the root growth inhibition in JA solutions (Staswick et al. 1992). In general, the regulatory effect of JA through auxin on LR formation is the net result of two competing mechanisms: JA promotes the *ANTHRANILATE SYNTHASE ALPHA SUBUNIT 1* (*ASA1*)-dependent auxin synthesis and JA reduces PIN-dependent auxin transport (Sun et al. 2009). JA is also integrated into auxin-mediated root meristem activity via *MYC2/JASMONATE INSENSITIVE1* (*MYC2*)-dependent repression of *PLETHORA* (*PLT*) expression (Chen et al. 2011). Another report, however, suggested that an auxin independent mechanism of the JA regulation of root development exists (Raya-González et al. 2012).

In rice, exogenous applications of MeJA on young seedlings retard the growth of roots and shoots. The lowest concentration of MeJA for the inhibition of rice seedling growth is 0.9 μM (Tsai et al. 1997) and 50% inhibition is observed at 5 μM of JA (Cho et al. 2007). Most differentially regulated proteins in rice roots effected by JA treatments belong to the functional categories of antioxidant, cellular respiration and, defense-related proteins (Cho et al. 2007). RICE SALT SENSITIVE3 (RSS3) was identified as a nuclear factor, which modulates the expression of JA-responsive genes and regulates root cell elongation. In a recent model, RSS3 binds with class-C basic helix-loop-helix (bHLH) transcription factors (TFs) and JASMONATE ZIM-DOMAIN proteins (JAZs) to form a ternary complex, as a result the JA-responsive genes are repressed when JA is absent. In the presence of JA, JAZ was degraded by the 26S proteasome. Thus, an unknown factor binds to the RSS3-bHLH complex, leading to the derepression of the bHLH-mediated transcription (Toda et al. 2013). KCl can partially relieve the MeJA inhibition of root growth because MeJA causes the conspicuous loss of KCl from the cell (Tsai et al. 1997). The JA inhibition of rice root growth can also be demonstrated by an endogenous increase in the JA level. For example, when pepper MAP kinase in rice is overexpressed, the endogenous JA accumulation is increased three times and the phenotype of root inhibition is similar to those of wild-type rice treated with 10 μM JA (Lee and Back 2005). The same phenotype of root growth inhibition is also observed in the rice NAC transcription factor gene *rim1* mutant, in which the expression levels of key genes involved in JA biosynthesis, such as *LOX*, *AOS2*, and *OPR7* are up-regulated because of the change in JA signaling (Yoshii et al. 2010). Additionally, it was found that the rice *ROOT MEANDER CURLING* (*OsRMC*) gene in the negative JA signaling pathway is involved in the development of the root system (Jiang et al. 2007).

#### Others

JA not only plays important roles in sterility, seed germination, and root growth in rice, but it also regulates other rice developmental processes, such as shoot growth, leaf senescence, spikelet development, photomorphogenesis, and gravitropism. For instance, exogenous applications of MeJA inhibit both shoot and root growth in rice (Tsai et al. 1997), the overproduction of JA in rice plants caused the lesion-mimic phenotype on the leaves (Liu et al. 2012b), and the senescence of rice leaves promoted by JA is associated with an increase in ET sensitivity rather than the ET level (Tsai et al. 1996). JA can determine the rice spikelet number and morphology through the JA signaling pathway or by mediating stress signals, subsequently affecting the grain yield (Cai et al. 2014; Kim et al. 2009). JA or its derivatives play an important role in the phytochrome-dependent inhibition of rice coleoptile elongation by light, indicating that JA is one of the hormones that regulate rice photomorphogenesis (Svyatyna and Riemann 2012) (see details in the section “JA-light crosstalk”). JA also modulates gravitropism, in an action mode opposite to that of auxin (Hoffmann et al. 2011; Gutjahr et al. 2005) (see details in the section “JA-auxin crosstalk”).

### JA-mediated abiotic stress responses in rice

Abiotic stresses, such as drought, salinity and adverse temperatures, have negative effects on plant growth and reproduction. When responding to these environmental changes, plants adjust the levels of endogenous phytohormones to activate biochemical and physiological pathways for adaptation. For example, ABA is the most studied hormone that responds to various environmental stresses, but other hormones, such as cytokinin, BR, and auxin, also participate in abiotic stress responses (Peleg and Blumwald 2011). Here, we emphasize the role of JA during environmental and abiotic stresses in rice. Under drought, salinity and cold stresses, the expression levels of many genes involved in JA biosynthesis were up-regulated and those genes involved in JA signaling were differentially modulated. At the same time, the JA level in rice was significantly increased compared with the non-stressed control (Moons et al. 1997; Tani et al. 2008; Du et al. 2013). This suggests that JA plays a critical role in regulating the responses and the adaptation of rice to diverse abiotic stresses. However, studies also showed that the JA level was decreased and JA biosynthetic genes were down-regulated under heat stress (Du et al. 2013), implying that different JA-regulating mechanisms may function under different abiotic stresses. There is a correlation between grain yield and MeJA level in rice (Cai et al. 2014; Kim et al. 2009). Increasing the MeJA level by overexpressing *Arabidopsis* jasmonic acid carboxyl methyltransferase (*AtJMT*) in rice resulted in a significant reduction in the grain yield. It was postulated that during the early stages of rice flower development under drought conditions, MeJA production is promoted by the induction of *OsJMT1*, a rice orthologue of *AtJMT.* The increased accumulation of MeJA activates the expression of *OsSDR*, which results in the high level of ABA biosynthesis. Drought stress also induces ABA accumulation directly, independently from MeJA. As a result, the accumulation of both MeJA and ABA affects spikelet development and subsequently causes a decrease in grain yield (Kim et al. 2009). Although the increased level of MeJA has a negative effect on rice grain production, JA accumulation may protect rice against salt stress. For example, exogenous applications of JA can ameliorate the performance of salt-stressed rice seedlings. This includes the recovery of many physiological properties, such as leaf water potential, lipopolysaccharide production, maximum quantum yield of Photosystem II, and calcium and magnesium uptake, when applying JA after saline exposure, and this recovery phenomena was more evident in the salt-sensitive cultivar than in the salt-tolerant cultivar (Kang et al. 2005). Additional assays also identified a higher endogenous accumulation of JA in salt-tolerant cultivars of rice than in salt-sensitive rice (Kang et al. 2005), implying that JA is involved in a protective mechanisms against salinity.

### JA-mediated rice resistance against pests and pathogens

Many reports indicated that JA plays critical roles in rice immunity against bacterial and fungal infections and damage by herbivores. In bacterial and fungal diseases, such as rice blight caused by *Xanthomonas oryzae* pv. *oryzae* (*Xoo*) and rice blast caused by *Magnaporthe grisea*, the exogenous application of JA is sufficient to induce rice resistance against infections, effectively reducing lesion symptoms (Yamada et al. 2012; Mei et al. 2006). Molecular assays revealed that the expression levels of several pathogenesis-related (*PR*) genes, including *OsPR1a*, *OsPR1b*, *OsPR2*, *OsPR5*, and *OsPR10*, are up-regulated in rice upon JA treatment (Yang et al. 2013), confirming that JA functions as an important signaling molecule in pathogen resistance. An analysis of transgenic rice plants that could accumulate high levels of endogenous JA through the overexpression of some key JA biosynthetic genes provided direct evidence of its function against microbial diseases. In rice, the overexpression of *OsAOS* significantly increases the JA level in the transgenic rice lines and the plants show enhanced resistance to the rice blight bacterium *Xoo* (Liu et al. 2012b) and rice blast fungus *M. grisea* (Mei et al. 2006). Similarly, the high accumulation of endogenous JA has a positive correlation with the activation of PR genes, such as *OsPR1a*, *OsPR3*, and *OsPR5*, in these transgenic rice plants (Mei et al. 2006). However, when *OsAOC*, encoding a functional allene oxide cyclase, is impaired in the rice mutants *coleoptile photomorphogenesis 2* (*cpm2*) and *hebiba*, enhanced fungal hyphal growth is observed compared with on wild-type (Riemann et al. 2013). The functional similarity of *AtAOS* and *OsAOS* in resistance mechanisms against pathogens was shown by the *Arabidopsis aos* mutant, which is more susceptible to the necrotrophic bacterium *Erwinia carotovora* ssp. *carotovora* strain WPP14 (*Ecc*) compared with wild-type and this phenotype can be complemented by potato *StAOS2* (Pajerowska-Mukhtar et al. 2008).

WRKY TFs play important roles in modulating JA signaling and many *WRKY* genes identified are JA responsive in *Arabidopsis* (Schluttenhofer et al. 2014). In the rice genome there are more than 100 *WRKY* genes, including *OsWRKY03*, *OsWRKY13*, *OsWRKY31*, *OsWRKY53*, *OsWRKY62*, *OsWRKY71*, *OsWRKY89* (Pandey and Somssich 2009), *OsWRKY45-1*, *OsWRKY45-2* (Tao et al. 2009), and *OsWRKY30* (Peng et al. 2012) that are associated with pathogen defense. The *OsWRKY45-1* and *OsWRKY45-2*-directed defense against *Xoo* is accompanied by an increased JA level, even though the molecular mechanisms of the rice-*Xoo* interactions modulated by these two genes are different (Tao et al. 2009). The expression levels of these two genes are directly regulated by OsWRKY13, which functions as a transcriptional repressor by binding to the promoter regions of the two genes. Thus, OsWRKY13 acts upstream of OsWRKY45s in their pathogen defense signaling pathway (Tao et al. 2009). Indeed, the activation of OsWRKY13 confers rice disease tolerance to *Xoo* and *M. grisea* (Qiu et al. 2007). OsWRKY30 also plays critical roles in the JA signaling pathway against rice pathogens. The overexpression of *OsWRKY30* in transgenic rice activates some JA biosynthetic genes, increases the JA accumulation level, and strengthens the rice plants ability to resist the rice sheath blight fungus *Rhizoctonia solani* and the blast fungus *M. grisea* (Peng et al. 2012)*.*

Jasmonoyl-L-isoleucine (JA-Ile) also plays an important role in JA signaling, and it modulates plant defenses against attacks by pathogens and insects in *Arabidopsis* and other dicots (Staswick et al. 1998; Kang et al. 2006). In rice, JA-Ile synthases, encoded by *OsJAR1* and *OsJAR2*, catalyze the JA-isoleucine conjugation to form JA-Ile. However, only the expression of *OsJAR1*, not that of *OsJAR2*, correlates with JA-Ile accumulation after blast attack (Wakuta et al. 2011), indicating that only *OsJAR1* is involved in pathogen defense. JA-Ile, as a primary and bioactive signal, stimulates the COI1 (a component of the E3 ubiquitin ligase SCF^COI1^)-mediated JAZ degradation, which is dependent on the 26S proteasome pathway, resulting in the activation of JA responsive genes that confer plant disease resistance (Katsir et al. 2008; Yamada et al. 2012). Indeed, the repression of *OsCOI1* expression or the overexpression of *OsJAZ8ΔC*, in which the Jas domain responsible for JA-dependent OsJAZ8 degradation was deleted, leads to the altered expression of JA-responsive genes and a reduced resistance to *Xoo* in transgenic rice plants (Yang 2009; Yamada et al. 2012). Additionally, the biosynthetic accumulation of monoterpene linalool is also regulated by OsJAZ8 in a JA-dependent manner, making the rice plants more resistant to *Xoo* (Taniguchi et al. 2014). These studies demonstrate that JA and JA signaling play critical roles in the regulation of pathogen defenses against bacteria and fungi.

JA or JA signaling also regulates rice plant defenses against some pests, such as nematodes and chewing herbivores. The exogenous treatment of rice shoots with MeJA triggers an intensive systemic defense response and activates the expression of some PR genes, including *OsPR1a* and *OsPR1b*, in the roots where the activities of the root knot nematode *Meloidogyne graminicola* is inhibited (Nahar et al. 2011). Although the mechanism behind how the non-expressor of pathogenesis-related genes1, *OsNPR1*, regulates JA biosynthesis remains unclear, *OsNPR1* knockdown rice plants exhibit enhanced levels of herbivore-mediated JA and JA-dependent resistance against infestation by the rice striped stem borer (SSB) (Li et al. 2013). However, contrasting roles for JA in controlling chewing and phloem-feeding herbivores were revealed by analyzing chloroplast-localized type 2 13-lipoxygenase gene (*OsHI-LOX*) antisense silenced rice plants (*as-lox* plants). Although the suppression of *OsHI-LOX* in rice decreases the SSB-induced JA accumulation and makes the rice more susceptible to SSB larvae, the *as-lox* rice plants exhibited an enhanced resistance to the rice brown planthopper (BPH) *Niaparvata lugens* (Zhou et al. 2009). Similar results were also obtained by studying rice mitogen-activated protein kinase (*OsMPK*) silenced plants. In these rice plants, the reduced elicited level of JA correlates with the improved performance of SSB larvae, but does not produce any positive effects on BPH (Wang et al. 2013), indicating that JA-related defense mechanisms are more complex than expected.

Recently, it was found that Mediator Complex Subunit25 (MED25/PFT1) mediates JA signaling and plays important roles in resistance to necrotrophic fungal pathogens. The Mediator Complex builds a bridge between TFs and RNA Pol II so that RNA Pol II can interact with adjacent TFs. *PHYTOCHROME AND FLOWERING TIME1* (*PFT1*) encodes the MED25 subunit of the plant Mediator. In *Arabidopsis*, MED25/PFT1 is necessary for JA-related defense gene expression, thus conferring resistance to the leaf-infecting fungi *Alternaria brassicicola* and *Botrytis cinerea*. However, *pft1* mutant plants show increased resistance to the root infecting hemibiotroph, *Fusarium oxysporum*, accompanying the significant attenuation of the expression of some JA-responsive genes, suggesting that MED25/PFT1 mediates *F. oxysporum*-induced disease progression through JA signaling (Kidd et al. 2009). Functions of MED25 may be conserved in monocots as TaPFT1, a wheat homologue of PFT1, is able to complement the *pft1*/*med25* mutation in *Arabidopsis* (Lyons et al. 2013). However, the role of rice OsPFT1, to our knowledge, is currently unknown. The rice homologue of *AtMed25_1* displays similar transcript levels in the reproductive and vegetative stages as found in *Arabidopsis* (Mathur et al. 2011). Therefore, studies on OsPFT1/MED25 will mark a new insight into JA-related signaling and defense responses in rice.

The antagonistic functions of the AOS and HPL branches in the oxylipin pathway should also be mentioned. These two branches generate JA and GLVs, respectively. Depletion of the expression of *OsHPL3* by mutation leads to the reduced level of E-2-hexenal, but the dramatic overproduction of JA, indicating that the disruption of the HPL pathway channels the substrates toward the JA pathway. This rice plant also showed the activation of JA-mediated defense responses in the resistance to the T1 strain of the bacterial blight *Xoo* is enhanced (Liu et al. 2012b). Studies on the *OsHPL3* rice mutant also revealed the differential defense regulatory roles of JA and GLVs, as the loss of *OsHPL3* function confers more resistance to the chewing herbivore SSB but more susceptibility to the phloem-feeding herbivore BPH. Indeed, OsHPL3 provides plants with resistance against BPH; however, it makes plants more susceptible to SSB and *Xoo* (Tong et al. 2012). Overall, the JA and HPL pathways are competing branches that provide plants with different defensive responses.

### JA crosstalk with other hormones in rice

#### JA-SA crosstalk

Previously, it was thought that JA and SA acted antagonistically upon pathogen infection and pest invasion throughout the plant kingdom. The antagonistic interaction of these two hormones can also be observed in rice plants. For example, in the OsWRKY13-mediated rice defense against bacterial blight and fungal blast, OsWRKY13 activates the SA-dependent signaling pathway and suppresses the JA-dependent signaling pathway. As a result, the expression of hormone-specific defense-related genes is differentially regulated (Qiu et al. 2007); In *Arabidopsis*, NPR1 regulates SA and JA antagonistically in response to invasion by *Pseudomonas syringae* pv *tomato* DC3000 (Spoel et al. 2003). The same cross-communicating signaling pathway is also conserved in rice plants, as the augmentation of the SA signal transduction pathway by OsNPR1 represses JA signaling pathway (Yuan et al. 2007). Further, JA and SA function antagonistically in OsPLD-mediated defense responses upon a SSB infestation of rice (Qi et al. 2011). However, synergistic JA-SA crosstalk was also found in rice. In the OsHPL3-mediated oxylipin pathway, JA and SA signaling were synergistically augmented in rice defenses against pathogens (Tong et al. 2012), although how SA signaling was activated remains to be elucidated. Therefore, the fine-tuning of overlapping signaling JA and SA pathways may be crucial for plant-specific defense responses.

#### JA-GA crosstalk

GA and JA are considered to act antagonistically to adjust the balance of energy allocation between growth and defense. Upon pathogen or insect invasion, JA facilitates the interaction between the JA receptor COI1 and JAZ proteins, the repressors of JA-responsive genes. Consequently, the JAZ proteins are degraded through polyubiquitination, resulting in the initiation of JA defense responses. As JAZ proteins also inhibit DELLA-PIF interactions, a crucial step in GA signaling, the degradation of JAZ repressors strengthens the repression of PIF TFs by DELLA proteins, thus GA-promoted growth is suppressed. However, when JA signaling is down-regulated, JAZ proteins can effectively interfere with the DELLA-PIF interaction, allowing the release of the PIF TFs, which in turn promotes GA-mediated growth (Yang et al. 2012). Indeed, *OsCOI1* knocked-down transgenic rice plants exhibit similar phenotypes to rice plants overproducing GA, and MeJA treatment facilitates the rice DELLA protein’s SLR1 accumulation and delays GA-regulated SLR1 degradation in wild-type rice plants (Yang et al. 2012). In addition, GA serves as a virulence factor of *Xoo* and *Magnaporthe oryzae* (*Mo*), and SLR1 is closely related to JA- and SA-dependent disease resistance pathways in rice (Filipe et al. 2014).

However, some preliminary studies revealed that a synergistic regulation by JA and GA in rice may exist. Under salt stress, treatment of rice plants with JA induces a higher accumulation of GA_1_ than that in the non-JA-treated control (Seo et al. 2005). In rice coleoptiles, there is a temporally high accumulation of *thionin* transcripts just after germination, followed by a gradual decline, and the level becomes undetectable in 3 days. Interestingly, the expression patterns of *thionin* genes paralleled those of key genes responsible for the biosynthesis of GA and BR, as was the change in the endogenous JA level. Furthermore, JA treatments exhibited similar effects as GA and BR co-treatments in suppressing the reduction of the *thionin* transcript level in the post-germination period (Kitanaga et al. 2006). These results strongly suggest that JA and GA act synergistically in rice under specific environmental conditions or at a specific developmental stage.

#### JA-ET crosstalk

Generally, it was thought that the JA and ET defense pathways in plants play synergistic functions that counteract invasions by necrotrophic pathogens (Glazebrook 2005) and herbivorous insects (Howe and Jander 2008). However, recent studies revealed that the JA and ET pathways are involved in rice resistance against the obligate biotrophs, root knot nematodes (Nahar et al. 2011). The treatment of rice shoots with exogenous Me-JA and ethephon induced systemic resistance against *M. graminicola* in roots. Further investigations using ET-insensitive transgenic rice and JA mutant rice plants, in which the JA biosynthetic pathway is disrupted, as well as assays blocking JA or ET biosynthetic pathways through the exogenous application of JA or ET biosynthetic inhibitors, confirmed that ET-mediated systemic defenses to *M. graminicola* depends on the activation of the JA biosynthetic pathway. Indeed, treatments of rice shoots with ET induce high expression levels of genes responsible for JA biosynthesis and signaling in roots. These all indicate that a synergistic effect of JA and ET against biotrophic pathogens exists. Herbivore infestation experiments further revealed that *OsNPR1* plays a negative role in the crosstalk between JA and ET signaling, as knocking down the *OsNPR1* gene resulted in increased expression levels of the lipoxygenase gene *OsHI-LOX* and an ACC synthase gene *OsACS2*, as well as increased production of JA and ET, conferring rice plants more resistance against SSB (Li et al. 2013). Antagonistic actions between JA and ET, however, may occur when rice plants protect themselves from infection by bacterial *Xoo*. The knockout of a rice enhanced disease resistance 1 gene, *OsEDR1*, exhibits reduced expression levels of the ACC synthase gene family, a decreased production of ET, and an enhanced resistance against bacteria. However, the plants show an activation of JA- and SA-associated pathways, suggesting that *OsEDR1* regulates the antagonistic interaction between the ET pathway and the JA or SA pathway (Shen et al. 2011).

#### JA-auxin crosstalk

The interaction between auxin and JA has been well studied in *Arabidopsis*. It was recognized that auxin promotes plant growth, whereas JA has an opposite function. In general, the interplay of these two hormones occurs at the levels of hormone perception, transport, and homeostasis, or through the interactions of their related regulatory proteins (Perez and Goossens 2013). However, only a few events of JA-auxin crosstalk have been reported in rice. One example is the gravitropism of rice coleoptiles driven by these two hormones. During the gravitropic curvature, a redistribution of auxin occurs, resulting in an auxin gradient, which triggers the differential growth of the upper and lower flanks of coleoptiles. Interestingly, a gradient of JA just opposite to that of auxin is also established and this JA gradient contributes to differential growth inhibition during the tropistic bending, suggesting that JA and auxin function antagonistically in response to gravitational stimulation (Hoffmann et al. 2011; Gutjahr et al. 2005).

#### JA-BR crosstalk

As mentioned above, the expression patterns of *thionin* genes are correlated with GA, BR, and JA in rice coleoptiles just after germination, and BR and GA act synergistically with JA at this specific growth stage (Kitanaga et al. 2006). However, when rice plants are under attack by root-knot nematodes, BR and JA play antagonistic functions in response to the infection. For instance, the activation of the BR pathway in rice root systems makes the plants more susceptible to nematodes, whereas the exogenous foliar application of MeJA strongly suppresses the BR pathway in roots and activates the JA-dependent rice innate immunity against the pathogens. Additionally, the expression of JA biosynthesis and signaling genes are significantly up-regulated in BR-deficient mutants compared with in wild-type, confirming that BR and JA interact negatively in rice roots (Nahar et al. 2013).

#### JA-ABA crosstalk

The crosstalk between JA and ABA is complex, and either synergistic or antagonistic depending on the different regulatory mechanisms mediated by the two hormones. As mentioned above, when rice plants in the early stage of flowering suffer drought stress, the normal development of the spikelet was affected by the synergistic action between JA and ABA, resulting in a decrease in grain production (Kim et al. 2009). Another example of synergism between these two hormones is that treatment of rice plants with JA and ABA, which leads to similar expression patterns of *OsCOI1*, a key gene functioning as a JA signaling receptor, implying that the JA and ABA signaling pathways are overlapped. However, when rice plants are under migratory nematode attack or under saline stress, antagonism may occur between ABA and JA. A recent study revealed that the JA pathway is necessary in the rice defense against *Hirschmanniella oryzae*. However, ABA plays a negative role by antagonizing JA biosynthesis and the signaling pathway, thus making the rice plants more susceptible to the nematode (Nahar et al. 2012). ABA and JA may also interact negatively under saline conditions. It was reported that JA suppresses the ABA-mediated induction of *Oslea3* expression, and JA and ABA play antagonistic regulatory effects on the expression of the salt-induced gene *salT* (Moons et al. 1997).

### JA-light crosstalk

Plants not only capture light for photosynthesis, but also perceive specific light qualities and transmit these light signals to modify their growth and development in response to the environment. Light signals are converted to developmental cues mainly through the interactions between light and hormone signals. More and more evidence has revealed that phytohormones, such as auxin, JA, and GA, are involved in light/phytochrome-regulated growth. In recent years, the crosstalk between JA and light has attracted special interest (Kazan and Manners 2011). In *Arabidopsis*, various components of the JA pathway, such as COI1, JAZ, MYC2, and JAR1, were found to influence light-mediated responses. For instance, JAR1 acts as a jasmonate-conjugating enzyme. The *jar1-1* mutant, with the substitution of Phe for the conserved Ser-101 in the open reading frame of JAR1, exhibits a long-hypocotyl phenotype under weak far red light (Wang et al. 2011). Similarly, the coleoptiles of the rice *Osjar1* mutant grew longer than those of the wild-type under continuous far red light, indicating that interference of the JA pathway in rice results in an altered sensitivity to far red light (Riemann et al. 2008). Although it has long been recognized that red light induces inhibition of rice coleoptile elongation (Furuya et al. 1969), the elongation of coleoptiles was not inhibited by irradiation with red light in the mutant *hebiba* in which *OsAOC*, an early gene in JA biosynthesis pathway, is impaired (Riemann et al. 2003), indicating that JA is involved in the light-mediated inhibition of coleoptile elongation. Another study revealed that the JA content increases and the expression of a key gene responsible for JA synthesis, *OsOPR*, is activated in response to red light irradiation; however, the red light-induced expression of *OsOPR* was eliminated in *hebiba* (Riemann et al. 2003). This implies that the JA deficiency is associated with light-mediated phenotypes in this mutant.

In addition, the transcription of *OsAOS1*, another key gene in JA biosynthesis, is enhanced by red light in a phytochrome-mediated manner in rice seedlings (Haga and Iino 2004), revealing that phytochromes play key roles in the connection between light and the JA pathway. The involvement of phytochromes, such as PHYA and PHYB, in the JA-light crosstalk can also be demonstrated from the further assays on *OsJAR1* since the expression of this gene is down-regulated in both *phyA* and *phyB* rice mutants and completely eliminated in the *phyA phyB* double mutant (Riemann et al. 2008). Another study in *hebiba* revealed that the photodestruction of phyA is delayed in this mutant; however, exogenous applications of MeJA accelerate the phyA degradation upon light activation (Riemann et al. 2009). A differential display screen between *hebiba* and wild-type rice also led to the identification of a JA-induced gene, GER1, which is redundantly controlled by PhyA and PhyB under red light (Riemann et al. 2007). Blue light is also thought to interact with the JA pathway since it induces the expression of *OsAOS1* in a cryptochrome (*Oscry1a* and *Oscry1b*)-mediated manner, resulting in the inhibition of rice coleoptile growth (Hirose et al. 2006).

JA-light crosstalk may also play a role in the JA-mediated defense against blast fungi in rice. Evidence revealed that the *phyA phyB phyC* triple mutant exhibits an increased susceptibility to *M. grisea* due to the down-regulation of the JA-responsive defense gene *PR1b* (Xie et al. 2011).

## Conclusions

Although most discoveries concerning the JA pathway and JA functions are from studies of the dicotyledonous model plant *A. thaliana*, recent endeavors have revealed that the functions of JA in rice, an important crop for human beings, are versatile. In rice, it regulates flower development and fertility, seed germination, root system development, shoot growth, leaf senescence, spikelet development, photomorphogenesis, and gravitropism as well as mediating responses to abiotic and biotic stresses. In general, the JA pathway interacts with the pathways of other hormones to compose a complex regulatory network. Both synergistic and antagonistic crosstalk exists between JA and other hormones, indicating that the fine-tuning of the JA pathway is necessary in rice plants so that the proper physiological activities can be implemented. It should be noted that some of the reports on the JA pathway in rice are still preliminary and efforts should be made to identify new components involved in the JA regulatory pathway.
